# Trends in Sports-Related Upper Extremity Injuries Presenting to United States Emergency Departments: A Retrospective Analysis of National Injury Data

**DOI:** 10.3390/jcm14176208

**Published:** 2025-09-02

**Authors:** Matthew D. Ramey, Srivatsan J. Swaminathan, Auston R. Locke, Niklas H. Koehne, Christoph A. Schroen, Visweshwar G. Swaminathan, John J. Corvi, Salvatore Capotosto, Paul J. Cagle, Michael R. Hausman

**Affiliations:** 1 Oakland University William Beaumont School of Medicine, 586 Pioneer Dr, Rochester, MI 48309, USA; matthewramey@berkeley.edu; 2 Icahn School of Medicine at Mount Sinai, 1 Gustave L. Levy Pl, New York, NY 10029, USAniklas.koehne@icahn.mssm.edu (N.H.K.);; 3 Medical Faculty Heidelberg, Heidelberg University, 69117 Heidelberg, Germany; 4 School of Life Sciences, Arizona State University, Tempe, AZ 85281, USA; vswamin6@asu.edu

**Keywords:** sports medicine, upper extremity, injury, emergency medicine, epidemiology

## Abstract

**Background:** Across many sports, injuries to the upper extremity (UE) are prevalent due to falls on an outstretched hand (FOOSH) and overuse. This retrospective analysis aimed to characterize sports-related UE injuries in the United States (US) over the past decade, examining injury frequency, affected body part, diagnosis, and hospital disposition across age, sex, and sport. **Methods:** The National Electronic Injury Surveillance System (NEISS) was queried for sports injuries presented to US emergency departments (EDs) from 1 January 2014 to 31 December 2023. Patient demographics, injury site, diagnosis, and disposition were recorded. Annual injury trends were evaluated by linear regression. All statistical analysis was conducted using SPSS (version 30.0) software. **Results:** There were 1,330,108 nationally estimated (NE) UE sports injuries (47,371 NEISS Cases) that were presented to US EDs from 2014 to 2023. Linear regression revealed a significant decrease in annual injuries across the study period (β = −0.63, R^2^ = 0.40 *p* = 0.05). For many sports, including football, basketball, soccer, baseball, softball, wrestling, volleyball, hockey, and rugby, rates of UE injury decreased significantly during the study period. Fractures were the predominant diagnosis across all age groups, observed among 99.3% of patients. Football was the most common sport associated with UE injury in elementary to high school-age children, with basketball becoming more common in patients from 19 to 50 years old. Tennis-related injuries were the most common for patients above 50 years old. Across all sports, the most commonly injured body parts were fingers (33.5%), wrists (20.3%), and lower arms (17.3%). Notably, shoulder injuries accounted for 99.1% of all wrestling cases and 71.5% of all lacrosse injuries included in this study. **Conclusions:** Sports-related UE injuries decreased significantly from 2014 to 2023, with notable declines during the COVID-19 pandemic. The injuries included in this study varied depending on sport, age, and sex, allowing for the recommendation of specific interventions. While few injuries led to hospitalization, those that did typically involved lower arm injuries; further efforts to reduce these injuries will reduce the burden placed on US hospitals.

## 1. Introduction

Injuries to the upper extremities (UEs) are a common diagnosis among athletes in many sports. These injuries are often a result of falling onto an outstretched hand (FOOSH) and overuse [1]. Injuries that occur through FOOSH mechanisms commonly present as fractures to the wrist and lower arm [2,3]. Overuse injuries tend to affect athletes in throwing-dominant sports due to repetitive motions involving the shoulder and elbow joints [4]. Previous research has shown that UE injuries tend to be sport specific. For example, shoulder injuries are common among football and baseball players, with the term “little league shoulder” being created to describe chronic stress fractures of the proximal humeral physis among little league (4–16 year old) baseball players [5]. Furthermore, the sport one opts to play is often determined by both age and sex. To illustrate, previous studies have shown that with increased age, participation in team sports tends to decline [6,7]. Given this, it is likely that sports-related UE injuries vary depending on the age, sex, and preferred sport of the patient.

Sports-related UE injuries have been researched extensively, although often in a relatively limited scope. Several studies have examined the prevalence of UE injuries in a specific sport, including, but not limited to: football, baseball, soccer, tennis, gymnastics, snowboarding, skiing, and rock climbing [8,9,10,11,12]. Some studies have explored UE injuries across a range of sports; however, these studies tend to restrict their analysis to a specific age group, with adolescents being a common patient population [13,14]. To our knowledge, no study has examined UE injury trends across multiple popular sports following the COVID-19 pandemic, despite the frequency of UE injuries among athletes. With participation in sports rising once again, the distribution of UE injuries across different sports is an important knowledge gap to address.

The purpose of this study was to add to the literature by providing an up-to-date assessment of sports-related UE injury trends in injury frequency, affected body parts, diagnosis, and hospital disposition across age, sex, and sport. This analysis was accomplished using National Electronic Injury Surveillance System (NEISS) data. NEISS is a preferred database due to its ability to use sample data to extrapolate to the national level. This enables an accurate assessment of injury trends and characteristics across the United States (US). This database was used to assess the most commonly implicated sports, most common diagnoses, and regions of the UE typically impacted. This analysis allowed us to identify common cases associated with different sports and potentially suggest preventative measures for at-risk athletes. We hypothesized that shoulder injuries would be most common among throwing-dominant sports, such as baseball and softball, while wrist and lower arm injuries would be most common among soccer and basketball players due to the likelihood of FOOSH injury mechanisms. We also hypothesized that injuries in sports typically associated with leisure (i.e., golf) would be more prevalent among older athletes. Finally, we hypothesized that injuries would be higher among male athletes compared to female athletes.

## 2. Methods

### 2.1. Database

The Consumer Product Safety Commission’s NEISS database is a national de-identified, publicly accessible database. Data from NEISS is collected from visits to US emergency departments (EDs) from hospitals with varying size, geographic location, and patient population. By using sample weights assigned to each of these EDs, national estimates (NEs) can be calculated for injuries across the United States. Sample weights are assigned to cases based on the ED which reported the data to NEISS. NEISS categorizes EDs into groups based on size, geographic location, and patient population served (i.e., children’s hospitals). The weight that NEISS assigns to each category is based on how many similar EDs exist nationwide. Computer software then takes these weights into account when calculating frequencies of NE cases. Thus, the NEs are representative of the NEISS cases multiplied by their assigned weight.

### 2.2. Data Extraction

The NEISS database was queried for all UE injuries (NEISS body part codes: 30 (shoulder), 32 (elbow), 33 (lower arm), 34 (wrist), 80 (upper arm), 82 (hand), and 92 (finger)) from 1 January 2014 to 31 December 2023. Each case from the NEISS database included the date of visit, age, sex, race, injury diagnosis, body part injured, hospital disposition, and a brief narrative about the presenting injury.

### 2.3. Data Cleaning

The extracted data was cleaned using RStudio version 2023.12.0+369. The initial dataset was queried to identify the sports which were most commonly associated with UE injuries. These sports included: football, basketball, soccer, baseball, softball, wrestling, volleyball, ice hockey, tennis, lacrosse, golf, and rugby. All other sports, which comprised small percentages of the total number of cases, were excluded. No NEISS categories were combined (for example, we chose to keep “ice hockey” and “hockey” as unique sports) Each narrative was then reviewed to exclude injuries not sustained while directly participating in sports (i.e., “pt sustained fx after falling down stairs at a football game”). To do so, a separate dataset was created including cases in which two or more products were included. For example, a case in which a patient fell down stairs at a football game would include the codes “stairs” (NEISS code 1842) and “football” (NEISS code 1211). These cases were reviewed by two authors (MDR and SJS) with all conflicts being resolved by a third author (ARL). The aforementioned inclusion and exclusion criteria left 47,371 NEISS cases for analysis. Age was then categorized into one of six groups: “youth” (0–10 years), “middle school” (11–13 years), “high school” (14–18 years), “college” (19–23 years), “young adult” (24–30 years), “adult” (31–50 years), “late adult” (51–64 years), and “senior” (65+ years). These age groups were selected to account for patterns of sports participation during the life course. From ages 0–18, athletes often play sports in leagues determined by age, such as little league, middle school teams, high school teams, and National Collegiate Athletic Association (NCAA) leagues. Throughout adulthood, athletes are likely to participate in more recreational sports due to the lack of leagues available beyond professional sports [9]. Additionally, as athletes progress through their adulthood, less physically demanding, non-contact sports become more popular due to weakened joints and decreasing muscle mass [10].

### 2.4. Statistical Analysis

IBM SPSS Statistics Version 28.0 (Chicago, IL, USA) was used for data analysis. Descriptive statistics (reported in NEISS cases, NE, and associated percentages) were used when examining the injuries by sex, age, body part, diagnosis, and patient disposition. Linear regression evaluated annual trends in sports-related hand and wrist injury; year of injury was the independent variable and frequency of injury was the dependent variable. This study elected to utilize linear regression to assess the overall direction and magnitude of temporal trends. Given the focus was on long-term trends rather than short-term fluctuations, we did not examine seasonality. Pearson’s chi-squared analysis was used to assess sex-specific differences. Multiple comparison adjustments were not applied. The level of statistical significance was set at *p* < 0.05.

## 3. Results

**Figure 1:**
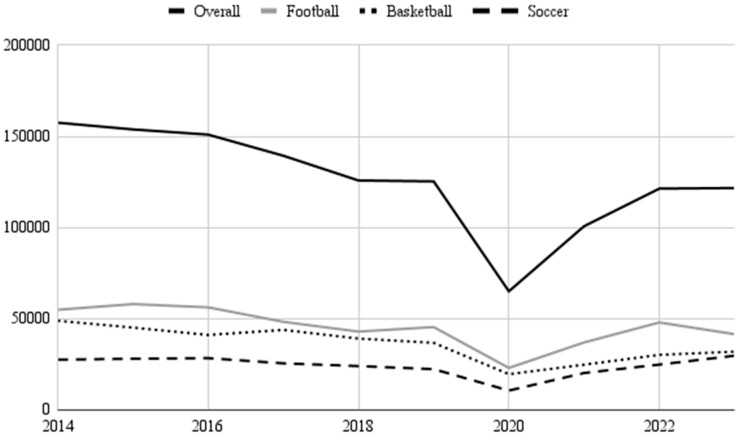
Frequency of sports-related upper extremity injuries vs. time.

There were 1,330,108 NE sports-related UE injuries (47,371 NEISS cases) presented to US EDs from 2014 to 2023. The frequency of sports-related UE injuries decreased significantly over the time period (β = −0.63, R^2^ = 0.40 *p* = 0.05), corresponding to an average annual decrease of 6097 injuries. All sports assessed in this study experienced a dip in injury frequency during the year 2020 (Figure 1). The three most common sports leading to UE injuries were football (NE: 454,984), basketball (NE: 361,188), and soccer (NE: 241,273). The temporal trends for these three sports are depicted in Figure 1.

An age-specific analysis revealed that a majority of sports-related UE injuries were sustained by the 14–18 (36.6%), 11–13 (32.6%), and 0–10 (17.0%) year-old age groups. Among age groups, football-related UE injuries were most prevalent among 0–10 (36.0%), 11–13 (38.7%), and 14–18 (37.6%) year-olds. Basketball-related UE injuries were most common among 19–23 (41.5%), 24–30 (33.4%), and 31–50 (29.3%) year-olds. Finally, tennis-related UE injuries were most often sustained among 50–65 (26.5%) and 65+ (43.3%) year-olds (Table 1).

Fingers were the most commonly injured body part overall (NE: 424,439). Finger injuries made up the highest proportion of football-, basketball-, baseball-, and softball-related injuries. Injuries to the wrist were most common during participation in soccer and tennis. The lower arm was most affected among golf- and volleyball-related UE injuries. Finally, shoulder injuries comprised the highest proportion of wrestling-, lacrosse-, and rugby-related UE injuries (Table 2).

**Figure 2:**
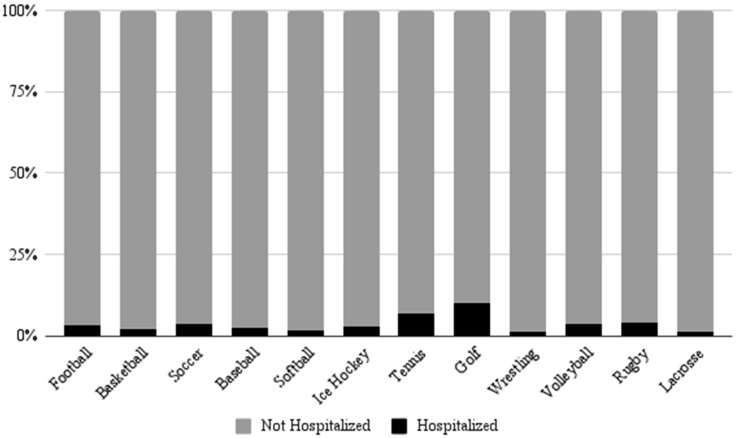
Percent of injuries requiring hospitalization versus injuries not requiring hospitalization by affected body part.

An analysis of patient disposition revealed that only 3.3% of patients in this study required hospitalization, with the remaining 96.7% of patients being treated and released or leaving without being seen. Across all cases involving hospitalization, 99.9% were due to fractures. Patient disposition by body part revealed that injuries to the elbow (10.2%), upper arm (9.9%), and lower arm (8.2%) had the highest proportions of hospitalization cases (Figure 2). Notably, nearly a majority (44.8%) of hospitalizations observed in this study were due to lower arm injuries.

**Figure 3:**
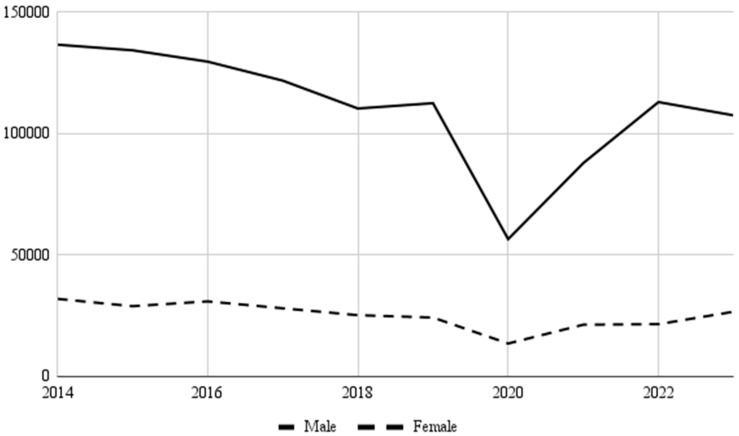
Injury prevalence among males and females over the study period.

**Figure 4:**
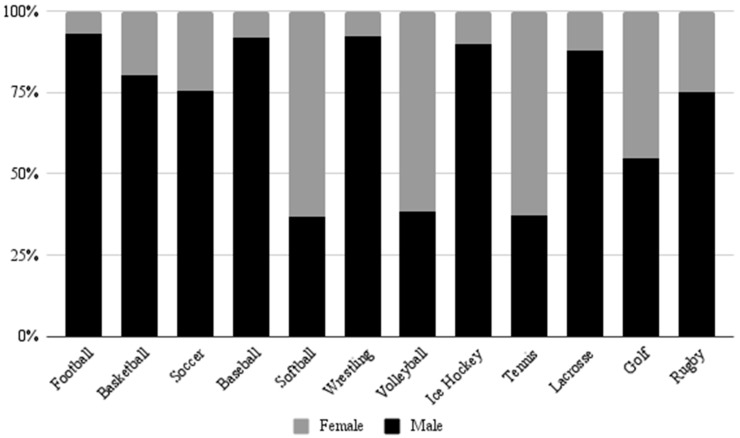
Percent of UE injuries by sport for males and females.

A sex-specific analysis of UE injuries revealed that males experienced more injuries than females (81.6% vs. 18.4%, *p* < 0.001) throughout the study period. Temporal injury trends for males and females are depicted in Figure 3. The most frequently injured body part was the finger for males (31.1%) and females (44.0%). For both males and females, fractures comprised the majority of UE injuries at 99.3% frequency for each gender. The sport with the highest proportion of male UE injuries was football (92.9%), while the sport with the highest proportion of female UE injuries was softball (63.4%) (Figure 4).

## 4. Discussion

This study assessed trends in sports-related UE injuries by examining injury frequency, affected body parts, diagnoses, and hospital disposition across age, sex, and sport using US injury data from 2014 to 2023. UE injuries decreased significantly over the study period. Sharp increases in injury frequency were observed between the years 2020 and 2024. These increases were likely due to increased participation in sports following the COVID-19 pandemic, as lifting shelter-in-place restrictions allowed athletes to return to participation in organized leagues [15]. It is interesting to note that previous studies have observed increased injuries among athletes returning to sport after prolonged breaks during the COVID-19 pandemic due to “detraining,” which caused decreased strength, endurance, and skill [16]. Across age groups, high school athletes (ages 14–18) made up the largest proportion of injuries. The sport that resulted in the highest frequency of UE injuries was football, followed by soccer and basketball. The majority of cases observed in this study were fractures. The results of the present study suggest the need for the implementation of improved regulations and protective gear to avoid fractures, specifically among the three aforementioned sports.

An age-specific analysis revealed that football-related UE injuries were most common among children, middle schoolers, and high schoolers. Among college athletes and adults, basketball contributed to the most UE injuries. Finally, late adults and seniors most frequently sustained UE injuries while participating in tennis. Previous studies have reported similar trends to those observed in this study [7,17,18,19]. These findings are likely due to the popularity of certain sports among different age groups. Many young athletes play football throughout middle and high school but often stop in college due to the limited number of opportunities to continue at the collegiate and professional level. Research from the NCAA estimated that only 7.5% of high school football players make it to the collegiate level, and of these players, 1.5% make it to a professional level [20,21]. After high school, the lack of opportunities to play at the competitive level causes many to instead participate in recreational athletics. This makes sports such as basketball—which require less equipment and a smaller playing area—more popular than sports such as football. National Health and Nutrition Examination Survey (NHANES) data shows that among individuals 18 and older, basketball participation rates are higher than that of football [22]. This shift may explain the prevalence of basketball-related UE injuries among older age groups. Among older adults and seniors, sports such as tennis and golf are more popular [23]. This also aligns with our findings, as tennis-related UE injuries were most common among late adults and seniors. Furthermore, golf-related UE injuries were the second-most common occurrence in seniors.

Injuries to the finger were most common overall and were the most common body part injured during participation in: football, basketball, baseball, and softball. While the mechanism of injury was not accounted for in the present study, findings from previous studies can provide useful insight into potential mechanisms; however, it should be noted that all proposed mechanisms for injuries are hypothetical. In football, a majority of injuries to the finger have been attributed to the high rate of contact, specifically during tackling, blocking, and FOOSH following player contact [24,25]. In each of these sports, play relies heavily on catching and throwing. These actions commonly result in injuries to the finger due to forceful hyperextension or direct blows, which may explain the prevalence of finger injuries among these sports [26]. Unfortunately, protective gear for the finger is likely to impede an athlete’s ability to catch and throw, and thus further studies on protective interventions that do not substantially impact play are necessary. One potential protective measure is taping the two ulnar digits at the proximal interphalangeal (PIP) joint, although there is a paucity of studies assessing the efficacy of this intervention [24]. Among volleyball athletes, injuries to the lower arm comprised a majority of affected body parts. Previous studies have attributed these injuries to impacts with the floor following diving and repetitive microtrauma due to spiking and digging [27,28]. The addition of protective equipment for the wrist and lower arm may be a useful intervention to improve volleyball player safety, although no studies have examined such an intervention and its impact. Notably, shoulder injuries were especially common during participation in wrestling, lacrosse, and rugby; furthermore, nearly all the wrestling injuries observed in this study involved the shoulder. Several studies have noted a high prevalence of shoulder injuries among wrestlers, even among studies not restricted to the UEs [29,30,31]. The main mechanisms of injury for these shoulder injuries are due to forced movement outside the normal range of motion and impact on the wrestling mat [29]. The prevalence of shoulder injuries in lacrosse are also in agreement with previous literature, and are typically the result of collisions with other players or the playing surface; however, collisions are typically observed among male athletes, as female lacrosse leagues are non-contact [32,33]. While less shoulder injuries were observed among rugby players compared to the two aforementioned sports, prior research indicates that rugby athletes are still at risk for shoulder injuries. Willigenburg et al. found that among a cohort of collegiate football and rugby players, rugby player’s risk of shoulder injury was greater than four times that of football players [34]. This is likely due to the fact that rugby players do not wear protective equipment while football players do. Shoulder injuries in rugby share similarities to those observed in both wrestling and rugby, with the main mechanisms reported in the literature being collisions, forced rotation during scrums—a play which involves players wrestling for control of the ball—and tackles [35,36]. The introduction of padding for the shoulders in wrestling and rugby may decrease collision-induced shoulder injuries, while proper stretching and post-play management may decrease shoulder tissue injuries across all three sports.

Hospitalization rates among patients in this study were low, with only 3.3% of patients requiring hospitalization. However, differences in hospitalization were observed among affected body parts, which offers valuable insight into the burden that certain injuries place on hospital systems. Injuries to the elbow, lower arm, and upper arm had the highest proportion of hospitalizations compared to other regions of the UEs. Furthermore, among all hospitalizations observed in this study, the majority were due to injuries to the lower arm, and another quarter were due to injuries to the wrist. Approximately 99.8% and 99.4% of all lower arm and wrist injuries, respectively, were fractures. This can be interpreted as fractures to the lower arm and wrist causing a disproportionate burden to orthopedic clinics nationwide. Although mechanisms of injury were unable to be examined in this study, some hypothetical explanations for these injuries are wrist hyperextension and FOOSH. These mechanisms are common across multiple sports, such as: football, basketball, volleyball and rugby [37,38]. Other common mechanisms include direct trauma as well as stress experienced while throwing or swinging [39]. Possible interventions to counteract these injuries are varied. Specifically, athletes should be taught how to properly fall to minimize the risk of injury. Additionally, contact sports should continue establishing and enforcing strict guidelines prohibiting unnecessary, dangerous contact. It may also be beneficial to employ more rigorous safety measures, such as wrist guards, to minimize UE injury.

A sex-specific analysis revealed that the most common diagnoses for both males and females were fractures to the finger while playing football and basketball, respectively. It is important to note that some sports, such as hockey and lacrosse, have different rule sets for male and female players. For example, no contact is allowed in women’s lacrosse, while in women’s hockey, no checking is allowed. These rules may explain some of the findings observed in this study. Specifically, 90.0% of total hockey injuries occurred in men’s hockey, and 87.8% of total lacrosse injuries occurred in men’s lacrosse. These statistics highlight the large contribution of direct trauma-related injuries to hockey and lacrosse injuries. This further demonstrates the importance of preventing dangerous contact.

## 5. Limitations

This analysis of UE injuries was limited by the inability to characterize injury mechanisms for each case. Additionally, while some of the included narratives from NEISS mention protective gear worn by the patient, this is not true of all narratives. Because of this, information on the presence or absence of protective gear was also unable to be included. While we made recommendations for protective gear implementation, further studies are needed to confirm the efficacy of these interventions. NEISS also only assesses injuries severe enough for patients to visit the ED, potentially excluding patients who managed injuries on their own or presented to other medical facilities, such as urgent and primary care visits. The distribution of injured body parts and diagnoses may differ among these cases, although this was unable to be accounted for in the present study. Furthermore, injury severity among included cases was not graded; thus we cannot make definitive claims regarding which injuries were the most severe beyond examining which injuries required further hospitalization. Finally, participation rates were not included in data collection, and we were unable to quantify the percentage of athletes sustaining a UE injury among all athletes for each sport.

## 6. Conclusions

This study demonstrated a significant decrease in sports-related UE injuries in the US, a promising finding regarding player safety. It should be noted that despite the overall decrease in injuries, an increase following the COVID-19 pandemic was observed, which may continue to rise as sports participation increases. Football, basketball, and soccer contributed to the most UE injuries included in this study. Injuries to the finger, wrist, and lower arm were most common, although affected body parts tend to vary by sport. While there were minimal hospitalizations observed in this study, a majority of hospitalizations were a result of lower arm injuries. The findings from this study, taken alongside observations cited in the previous literature, support the implementation of improved safety equipment as well as pre- and post-play stretching routines. Future research should attempt to characterize mechanisms of lower arm injury that lead to hospitalization.

## Figures and Tables

**Table 1:** Top three sports-related upper extremity injuries vs. age group.

	Age Group							
	Child (0–10)	Middle School (11–13)	High School (14–18)	College (19–23)	Young Adult (24–30)	Adult (31–50)	Late Adult (51–64)	Senior (65+)
Top Three Sports	1. Football (36.0%)	1. Football (38.7%)	1. Football (37.6%)	1. Basketball (41.5%)	1. Basketball (33.4%)	1. Basketball (29.3%)	1. Tennis (26.5%)	1. Tennis (43.3%)
	2. Bicycling (30.0%)	2. Basketball (27.1%)	2. Basketball (27.7%)	2. Football (23.3%)	2. Football (23.3%)	2. Soccer (18.5%)	2. Basketball (20.4%)	2. Golf (23.5%)
	3. Basketball (21.4%)	3. Soccer (18.2%)	3. Soccer (14.1%)	3. Soccer (17.0%)	3. Soccer (17.0%)	3. Football (17.2%)	3. Baseball (11.3%)	3. Softball (9.5%)

**Table 2:** Top three affected body parts by sport.

Football	Basketball	Soccer	Baseball	Softball	Ice Hockey	Tennis	Golf	Wrestling	Volleyball	Rugby	Lacrosse
1. Finger (35.6%)	1. Finger (45.2%)	1. Wrist (31.9%)	1. Finger (37.7%)	1. Finger (47.1%)	1. Shoulder (34.2%)	1. Wrist (45.6%)	1. Lower arm (24.7%)	1. Shoulder (99.1%)	1. Lower arm (62.2%)	1. Shoulder (46.5%)	1. Shoulder (71.5%)
2. Shoulder (18.7%)	2. Wrist (19.1%)	2. Lower arm (25.0%)	2. Lower arm (16.7%)	2. Wrist (16.7%)	2. Wrist (26.2%)	2. Lower arm (25.1%)	2. Wrist (23.7%)	2. Elbow (0.9%)	2. Elbow (15.6%)	2. Finger (24.7%)	2. Lower arm (17.3%)
3. Lower arm (16.6%)	3. Lower arm (14.3%)	3. Shoulder (16.3%)	3. Wrist (16.4%)	3. Hand (14.6%)	3. Lower arm (16.3%)	3. Shoulder (8.2%)	3. Upper arm (14.4%)	3. N/A	3. Shoulder (11.1%)	3. Wrist (12%)	3. Elbow (11%)

## Data Availability

The original data presented in this study is available in the NEISS Database at https://www.cpsc.gov/Research--Statistics/NEISS-Injury-Data (accessed on 13 August 2025).

## Abbreviations

ED	Emergency department
FOOSH	Fall on an outstretched hand
NCAA	National Collegiate Athletic Association
NEISS	National electronic injury surveillance system
NE	National estimate
NHANES	National Health and Nutrition Examination Survey
PIP	Proximal interphalangeal
UE	Upper extremity
US	United States
